# Time matters: CB2 receptor, developmental stress sensitivity and affective vulnerability

**DOI:** 10.3389/fcell.2026.1967345

**Published:** 2026-10-27

**Authors:** Abraham B. Torregrosa, Francisco Navarrete, María Salud García-Gutiérrez

**Affiliations:** 1 Instituto de Neurociencias, CSIC-Universidad Miguel Hernández, Alicante, Spain; 2 Red de Investigación en Atención Primaria de Adicciones, Instituto de Salud Carlos III, MICINN and FEDER, Madrid, Spain; 3 Instituto de Investigación Sanitaria y Biomédica de Alicante (ISABIAL), Hospital General Universitario Dr. Balmis, Alicante, Spain

**Keywords:** adolescence, affective disorders, CB2R, development, early-life stress

## Abstract

Early-life experiences are among the strongest predictors of vulnerability to emotional disorders, including depression in adulthood. Despite this association, the neurodevelopmental mechanisms underlying this link remain poorly understood. In this context, the endocannabinoid system—a neuromodulatory lipid signaling system in the brain—has garnered attention for its role in regulating stress responses and emotional behavior during development. Initially, most studies focused on the cannabinoid receptor type 1, but in recent years, an increasing number of studies have examined the role of cannabinoid receptor type 2 (CB2r) as a potential key modulator of brain response to environmental challenges during developmental states. This review aims to summarize current evidence on how adverse experiences during early life and adolescence influence CB2r in the brain, explore the connection between CB2r and the neuroimmune and behavioral alterations induced by these experiences, and evaluate how pharmacological and genetic studies support a role for CB2r in modulating brain responses to early adversity. Evidence from models of early-life stress, prenatal or perinatal drug exposure, adolescent stress, and recreational drug use indicates that CB2r expression is dynamically regulated by developmental stage, brain region, and biological sex. Moreover, pharmacological and genetic approaches have begun to provide functional evidence that modulation of CB2r may influence the neuroimmune and behavioral consequences of these developmental insults. Although further studies are needed to fully understand the complex role of CB2r, evidence to date suggests a potential involvement of this cannabinoid receptor in the affective and neuropsychiatric consequences of developmental adversity.

## 1 Introduction

Brain maturation is shaped by dynamic processes regulated by genetic and environmental factors (Krishnan and Nestler, 2010; Martin et al., 2009). This complex process begins during the prenatal period, characterized by intense neurogenesis and cellular differentiation, but extends well beyond birth, involving critical phases of circuit formation and refinement throughout early postnatal life and adolescence (Bale et al., 2010; Hoeijmakers et al., 2014). Accordingly, early life and adolescence represent sensitive developmental windows during which the brain is particularly susceptible to environmental modulation.

Exposure to adverse experiences during these critical developmental periods has been consistently associated with increased vulnerability to neuropsychiatric disorders later in life, including anxiety and depression, as well as substance use disorders (Gunnar et al., 2007; Howell and Sanchez, 2011; Browne et al., 1986; Bany-Mohammed et al., 2025). This increased vulnerability has been linked to long-lasting alterations in neuroendocrine and neurotransmitter systems, as well as disruptions in the maturation of brain circuits involved in emotional regulation (Naninck et al., 2015; Brunson et al., 2005; Gluckman et al., 2008; Gunn et al., 2013). Importantly, accumulating evidence suggests that these effects may be mediated, at least in part, by neuroimmune and neuroinflammatory mechanisms that shape stress responsivity across development (Catale et al., 2020; Calcia et al., 2016).

In recent years, the endocannabinoid system (ECS) has attracted increasing attention in this context. Growing evidence suggests that the ECS may contribute to fundamental processes in brain development, including neuronal differentiation, axonal guidance, synapse formation, and circuit maturation, particularly through cannabinoid receptor type 1 (CB1r) (Rodrigues et al., 2024; Song et al., 2021; Basavarajappa et al., 2009). More recently, attention has also turned to cannabinoid receptor type 2 (CB2r), which may contribute to the brain’s response to developmental challenges.

CB2r is widely expressed throughout the brain, from cortical areas to the cerebellum, including frontal and limbic regions, such as the hippocampus (HIP) and amygdala (AMY) (Garcia-Gutierrez et al., 2010; Gong et al., 2006; Van Sickle et al., 2005). More in detail, CB2r is expressed in multiple brain cell types, including neurons, astrocytes and microglia, and its expression appears to be dynamically regulated across development (Xi et al., 2025; Komorowska-Muller and Schmole, 2020; Grabon et al., 2024). Notably, CB2r has been identified in neural progenitor cells and in specific neuronal populations within the HIP (Palazuelos et al., 2006; Palazuelos et al., 2012; Li and Kim, 2015; Stempel et al., 2016; Visvanathar et al., 2022), as well as in human fetal astrocytes (Sheng et al., 2005), supporting its presence during early stages of brain development. In addition, its expression differs between pre-pubertal and adult stages, suggesting developmental regulation (Rodrigues et al., 2024; Zurolo et al., 2010).

CB2r modulates microglial proliferation and migration, as well as their functional phenotype, promoting a shift from pro-inflammatory to anti-inflammatory states and reducing the release of inflammatory mediators (Sharma et al., 2025; Grabon et al., 2023; Mecha et al., 2015; Meanti et al., 2025). Experimental evidence further supports a role for CB2r in modulating stress-induced neuroinflammatory responses and behavioral outcomes. Pharmacological activation of CB2r attenuates stress-induced inflammation and depressive-like behaviors, whereas genetic deletion of CB2r enhances neuroinflammatory responses and vulnerability to stress (Garcia-Gutierrez et al., 2010; Garcia-Gutierrez et al., 2025; Garcia-Gutierrez et al., 2013; Chen et al., 2024; Sahu et al., 2019). In addition, CB2r has been implicated in the regulation of neuronal proliferation and survival (Palazuelos et al., 2012; Onaivi, 2011), potentially through MAPK-ERK and Akt pathways (Haspula and Clark, 2020; Merighi et al., 2012). Its involvement in neuroimmune signaling, inflammatory responses, and synaptic plasticity has led to increasing interest in its potential contribution to the neurobiological adaptations triggered by developmental adversity.

Over the past decade, an increasing number of studies have examined how adverse experiences during early life and adolescence affect CB2r expression. Evidence from models of early-life stress (ELS), prenatal or perinatal drug exposure, adolescent stress, and recreational drug use indicates that CB2r expression is dynamically regulated by developmental stage, brain region, and biological sex. Moreover, pharmacological and genetic approaches have begun to provide functional evidence that modulation of CB2r may influence the neuroimmune and behavioral consequences of these developmental insults.

In this review, we summarize the current evidence on how developmental adversity, including ELS, prenatal or perinatal drug exposure, adolescent stress, and drug exposure, influences brain development and contributes to long-term behavioral alterations, with particular emphasis on CB2r. Specifically, we integrate current findings on how these adverse experiences influence CB2r expression, examine the available evidence linking CB2r to neuroimmune and behavioral alterations, and highlight pharmacological and genetic studies that examine the functional contribution of CB2r to the affective and neuropsychiatric consequences of developmental adversity.

## 2 Early-life adversity and CB2r

### 2.1 Developmental vulnerability to early-life adversity

Brain development during the early postnatal period is a dynamic and finely regulated process that underlies the initial formation and organization of neuronal circuits (Levitt, 2003; Jernigan et al., 2011). Although the basic architecture of the brain is largely established at birth, its maturation extends widely into the postnatal period, characterized by intense neurogenesis, neuronal migration, axonal growth and guidance, and the establishment of synaptic connections. This makes early life a critical window of heightened neuroplasticity and sensitivity to environmental influences.

During prenatal development, most neurogenesis occurs, producing the neuronal populations that will form the different brain regions (Kourosh-Arami et al., 2021). These neurons subsequently migrate to their final locations, contributing to the laminar organization of structures such as the cerebral cortex. In parallel, axonal growth and guidance establish the initial connections between brain regions, forming the basic scaffold of brain circuits (Accogli et al., 2020; Stiles and Jernigan, 2010).

Following birth, the brain undergoes an intense phase of reorganization. One of the most characteristic processes of this period is synaptogenesis, which involves the massive formation of synaptic connections, especially in brain regions closely related to emotional processing and cognition, such as the HIP, AMY, and prefrontal cortex (PFC) (Sorrells, 2024). This initial overproduction of synaptic connections supports high plasticity and adaptation to environmental inputs. Subsequently, synaptic pruning removes weaker connections while strengthening functionally relevant ones. This process helps refine neuronal circuits and relies on activity and experience (Xing et al., 2026; Huttenlocher et al., 1997).

Moreover, neurons undergo crucial structural changes, including dendritic growth and spine formation, which are essential for efficient synaptic communication. Concurrently, progressive myelination and maturation of neurotransmitter systems, including glutamatergic, GABAergic, dopaminergic, and serotonergic signaling, support the establishment of functional networks and the proper regulation of excitatory-inhibitory balance (Baumann et al., 2001; Larsen and Luna, 2018).

Importantly, interaction between the immune system and the central nervous system plays a key role during this period (Sakers et al., 2026; Mastenbroek et al., 2024). Microglia regulate neurogenesis, synaptic pruning, and the maturation of neuronal circuits, acting as sensors of the local environment and translating external signals into changes in neuronal circuit organization.

Overall, early brain development can be conceptualized as a process that combines the initial formation of neural circuits with their progressive refinement. This heightened plasticity enables environmental adaptation but also increases vulnerability, which explains how adverse life experiences, such as early stress, can induce profound and long-lasting alterations in brain structure and function (Tottenham, 2014; Fox et al., 2010).

ELS is not only associated with transient alterations but may actively reprogram cognitive and emotional brain networks during development. In this context, the quality and predictability of early environmental signals appear to be critical determinants of long-term outcomes (Davis et al., 2017). In mammals, including humans, rodents, and monkeys, maternal input is proposed as one of the most significant influences on the environment experienced during development. Disrupted or fragmented patterns of early-life sensory input have been proposed to induce chronic stress and interfere with the maturation of neural circuits underlying emotional regulation (Chen and Baram, 2016). In accordance, most animal models of ELS have focused on disrupting maternal interactions. In rodents, maternal separation is a well-established model of ELS that reproduces specific aspects of disrupted maternal care and mother-offspring interactions relevant to adverse experiences in humans. This model leads to both immediate and long-lasting alterations in emotional reactivity and increased vulnerability to stress-related disorders (Champagne et al., 2003). These behavioral outcomes are accompanied by neuroendocrine alterations, including dysregulation of the hypothalamus-pituitary-adrenal (HPA) axis, elevated stress hormone levels, and activation of pro-inflammatory pathways (Guijarro et al., 2007).

At the neural level, ELS disrupts the maturation and structural integrity of brain circuits involved in socioemotional processing, particularly within cortico-limbic networks such as the AMY, HIP, and PFC (Huang, 2014; Teicher et al., 2016). These alterations include reduced volume of stress-sensitive brain regions, impaired synaptic function, and changes in dendritic architecture and spine dynamics, which may ultimately compromise synaptic plasticity and cognitive function (Hulshof et al., 2011; Huot et al., 2002). In this context, abnormal maternal care has been shown to increase the number and function of excitatory synapses onto stress-sensitive hypothalamic neurons, contributing to sensitization of central components of the neuroendocrine stress system and increased vulnerability to subsequent stress (Jung, 2025; Martinez et al., 2026). In addition, developmental processes such as myelination, which are highly dynamic during early life, are particularly susceptible to environmental influences, including stress (Teissier et al., 2020; Antontseva et al., 2020; Nichols et al., 2025; Abraham et al., 2023).

A growing body of evidence indicates that these long-term effects are mediated, at least in part, by neuroimmune mechanisms. Exposure to ELS alters microglial numbers, morphology, and function in a region-, age-, and sex-dependent manner (Johnson and Kaffman, 2018; Zhang et al., 2025). For instance, prenatal stress increases microglia density in the developing brain, an effect reported from early developmental stages through adolescence and adulthood, particularly in regions such as the HIP and PFC (Diz-Chaves et al., 2012; Smai et al., 2024). These changes are accompanied by shifts in microglial morphology, with a predominance of ameboid forms during early stages, followed by a transition to ramified phenotypes after birth and during adolescence, and a shift toward more reactive profiles in adulthood.

Crucially, ELS has been proposed to induce long-lasting changes in microglial function, often referred to as microglial priming, in which these cells exhibit altered reactivity to subsequent challenges. This persistent neuroimmune reprogramming may contribute to exaggerated inflammatory responses and impaired circuit function later in life, thereby increasing vulnerability to stress-related affective disorders (Catale et al., 2020). Microglia activation has been associated with alterations in neurogenesis and synaptic plasticity, suggesting a mechanistic link between ELS, neuroimmune dysregulation, and affective vulnerability (Fang et al., 2023).

In addition to ELS, prenatal and early postnatal exposure to psychoactive drugs is a critical form of early adversity that alters brain maturation (Fisher et al., 2011). Despite several factors influencing the impact of these drugs, consumption during these developmental windows often produces long-lasting effects on brain structure and function, including alterations in brain maturation, structural connectivity, cell migration, and neurotransmitter systems (Kim et al., 2017; Ross et al., 2015; Stankovic and Colak, 2022). More specifically, fundamental cellular processes such as neurogenesis, neuronal migration, and synaptic pruning have been altered. Furthermore, this exposure compromises the structural and functional connectivity of cortical and subcortical regions (Shah et al., 2025). Within the framework of early adversity, psychoactive drugs induce long-term cognitive and behavioral deficits and have been associated with reduced adaptive brain plasticity, increasing vulnerability to subsequent environmental stressors throughout childhood and adolescence (Shah et al., 2025; Eiden et al., 2023; Buckingham-Howes et al., 2012; Buckingham-Howes et al., 2016).

In this context, CB2r has gained attention as a potential modulator of the neurobiological consequences of early-life adversity, particularly given its involvement in neuroimmune and inflammatory processes.

### 2.2 Evidence for CB2r involvement in early-life adversity-induced vulnerability

Exposure to early-life adverse experiences has been shown to influence CB2r expression and function and has been associated with long-lasting alterations in neuroimmune processes, synaptic plasticity, and emotional behavior. Available evidence indicates that ELS dynamically regulates CB2r in a brain region-, sex-, and developmental stage-dependent manner. Rather than representing a transient response to early adversity, these alterations persist throughout development and are associated with the emergence of stress-related behavioral abnormalities.

Experimental models of ELS indicate that CB2r expression is dynamically remodeled from the earliest stages of development. Increased CB2r immunoreactivity has been detected in the HIP shortly after maternal deprivation, indicating that alterations in CB2r emerge rapidly following early adverse experiences (Suarez et al., 2009). Rather than inducing a uniform increase or decrease in receptor expression, ELS appears to reshape CB2r expression according to developmental stage, biological sex, and brain region. Accordingly, maternal separation increased CB2r expression in parvalbumin-positive interneurons of the PFC during early adolescence, whereas receptor expression declined later in adolescence, coinciding with the emergence of depressive-like behaviors and emotional deficits (Andersen, 2024). Likewise, maternal deprivation induced sex-dependent changes in CB2r protein expression during adolescence, with increased hippocampal and prefrontal levels in females but reduced expression in males, accompanied by depressive- and psychotic-like phenotypes (Marco et al., 2014). These alterations appear to extend into adulthood, as increased Cnr2 expression has also been reported in the medial prefrontal cortex (mPFC) of adult females exposed to limited bedding and nesting, together with sex-dependent behavioral alterations (Portugalov et al., 2022). Collectively, these findings indicate that ELS induces a long-lasting and context-dependent remodeling of CB2r that is associated with emotional and behavioral dysfunction.

While descriptive studies indicate that ELS alters CB2r expression across development, functional approaches have begun to clarify whether these alterations actively contribute to the behavioral consequences of early adversity. Available evidence suggests that CB2r activation may attenuate neuroimmune alterations induced by early adversity, particularly through the regulation of microglial inflammatory responses. Maternal separation reduced MARCH7 expression while promoting NLRP3 inflammasome activation and IL-1β signaling in cortical microglia, resulting in a persistent pro-inflammatory state associated with depressive-like behaviors. Pharmacological activation of CB2r with HU-308 partially restored MARCH7 expression, attenuated inflammasome activation, and reduced neuroinflammatory signaling. Furthermore, both HU-308 treatment and CB2r overexpression in the prelimbic cortex through lentiviral delivery prevented the development of anhedonia and depressive-like behaviors during adolescence. Together, these findings provide functional evidence that enhancing CB2r signaling may attenuate the long-term neuroimmune and behavioral consequences of ELS, thereby promoting resilience to the long-term consequences of early-life adversity (Andersen, 2024).

Beyond ELS, available evidence indicates that prenatal or perinatal drug exposure induced alterations in CB2r. Perinatal exposure to dronabinol induced long-lasting emotional and cognitive alterations, together with changes in Cnr2 expression in the HIP and AMY and other components of the ECS (Navarro et al., 2024). Similarly, perinatal alcohol exposure (PAE) increased hippocampal Cnr2 expression in both male and female offspring, accompanied by persistent emotional and cognitive alterations (Gasparyan et al., 2023). Interestingly, early and chronic treatment with cannabidiol (CBD) attenuated several of these behavioral alterations and partially normalized Cnr2 expression in males, whereas this effect was not observed in females (Gasparyan et al., 2023). Besides, PAE increased Cnr2 expression in the nucleus accumbens, particularly in males. These changes were associated with alterations in gut microbiota composition and behavioral outcomes, further suggesting that CB2r-related adaptations following PAE may occur within broader gut-brain and behavioral alterations (Navarrete et al., 2025).

Overall, the available evidence suggests that environmental challenges during early development can lead to long-lasting and context-dependent changes in CB2r, with notable differences depending on development stage, sex, and brain region, which are linked to emotional and behavioral issues (Figure 1, Table 1).

**FIGURE 1:**
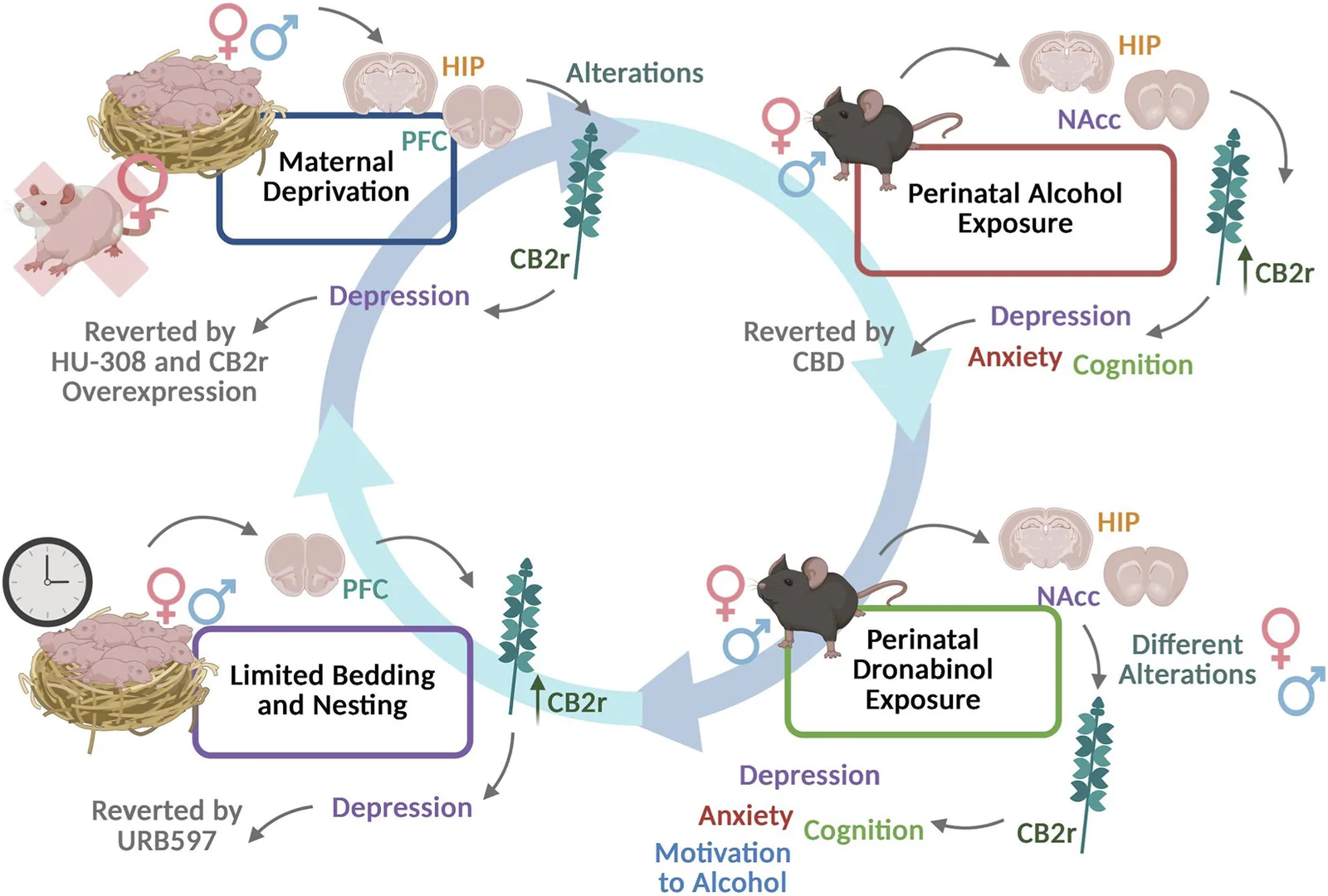
Schematic representation of the main findings supporting the involvement of CB2r in the consequences of early-life adversity. This figure summarizes the main CB2r alterations reported in experimental models of early-life stress and prenatal or perinatal drug exposure, and the associated behavioral and neurobiological outcomes. The effects of pharmacological and genetic interventions evaluated in these models are also represented. HIP: hippocampus; NAcc: nucleus Accumbens; PFC: prefrontal cortex. ↑ Indicate increased expression of CB2r.

**TABLE 1:** CB2r receptor alterations and functional evidence following early adversity.

Model	Species/Strain	Sex	Developmental period/Assessment	Brain region	CB2r-related findings	Behavioral outcomes	Pharmacological/genetic manipulation	Molecular outcomes	Behavioral outcomes	Refs.
Maternal deprivation (24h, PND9-10)	Rat/Wistar	M/F	Neonatal	HIP	Altered CB2r immunoreactivity; sex-dependent effects	_	_	_	_	Suarez et al. (2009)
Maternal separation (PND2-20)	Rat/Sprague-Dawley	F	PND25, PND40, PND100	Prelimbic PFC	↑CB2r PV neurons (PND25) ↓ CB2r PV neurons (PND40)	Depressive-like phenotype at PND40	HU-308 (2.5 mg/kg, i.p.; PND30, 32, 34, 36 and 38) CB2r-overexpressing lentivirus (bilateral, prelimbic cortex, PND33)	CB_2_r enhancement attenuated inflammatory alterations (MARCH7, NLPR3)	HU-308 and CB_2_r overexpression reduced/prevented depressive-like behaviors	Andersen (2024)
Maternal deprivation (24h, PND9)	Rat/Wistar	M/F	Adolescence	HIP, PFC and other regions	Sex- and region-dependent CB_2_r changes	Depressive- and psychotic-like alterations	_	_	_	Marco et al. (2014)
Limited bedding and nesting	Rat/Sprague-Dawley	M/F	Adulthood	mPFC	↑ Cnr2 in F	Depressive-like phenotype	FAAH inhibition with URB597 (0.4 mg/kg, i.p.; PND45-60)	URB597 reversed changes in CB_2_r	URB597 reversed several behavioral alterations	Portugalov et al. (2022)
Perinatal dronabinol exposure (10 mg/kg/12h; p.o.) (GD5-PND21)	Mouse/C57BL/6J	M/F	PND28-60	HIP, AMY	↑ Cnr2 in HIP and AMY in M ↑ Cnr2 in HIP in F	Anxiety- and depressive-like behaviors, cognitive impairment, reduced sensorimotor gating and ↑motivation/alcohol consumption	_	_	_	Navarro et al. (2024)
Perinatal alcohol exposure (PAE) (GD7-PND21)	Mouse/C57BL/6J	M/F	Adolescence/Adulthood	HIP	↑ Cnr2 in M/F	Anxiety-, depressive-like and cognitive alterations	CBD (30 mg/kg/day, i.p.; PND21-onwards)	CBD tended to normalize Cnr2 in M CBD improve molecular alterations induced by PAE (HPA-related genes, neuroplasticity and glutamatergic markers)	CBD improved most behavioral outcomes, with sex-dependent effects	Gasparyan et al. (2023)
PAE (GD7-PND21)	Mouse/C57BL/6J	M/F	Adolescence/Adulthood	NAcc	↑ Cnr2 in M, with a larger effect than in F Cnr2 associated with microbiota/behavioral measures	Anxiety-, depressive-like and cognitive alterations	CBD (30 mg/kg/day, i.p.; PND21-onwards)	CBD normalized brain molecular and gut microbiota alterations	CBD normalized alcohol motivation in F, and anxiety- and depressive-like-behaviors in M and F	Navarrete et al. (2025)

PND, postnatal day; PAE, perinatal alcohol exposure; M, male; F, female; HIP, hippocampus; PFC, prefrontal cortex; AMY, amygdala; NAcc, nucleus accumbens; CBD, cannabidiol; PV, parvalbumin; ↑: increase; ↓: decrease; HPA, hypothalamic-pituitary-adrenal axis; GD, gestational day; p.o: intragastric administration; i.p.: intraperitoneal administration.

## 3 Adolescent adversity and CB2r

### 3.1 Developmental vulnerability during adolescent adversity

Adolescence, spanning approximately from 10–11 to 18 years of age, is marked by significant neurodevelopmental changes involving the refinement and maturation of pre-existing neuronal circuits (Griffin, 2017). During this period, brain development is primarily driven by synaptic pruning and myelination, with the strengthening and optimization of brain neuronal networks, particularly in the frontostriatal and cortico-limbic circuits (Selemon, 2013).

Synaptic pruning is particularly prominent during adolescence, notably in regions underlying higher-order cognitive functions such as the PFC, where a substantial portion of excitatory synapses is eliminated (Chechik et al., 1999; Spessot et al., 2004; Rakic et al., 1994). This process improves brain function and efficiency (Selemon, 2013) and contributes to the reorganization of brain connections toward patterns typical of adulthood, thereby influencing neuronal plasticity in later life stages (Spear, 2013). In parallel, increased myelination (Cicchetti and Donald, 2006) improves the processing speed (Spear, 2013; Counotte et al., 2011) and the reliability of neuronal transmission, further supporting functional integration across brain regions (Geier and Luna).

These structural changes are accompanied by the maturation of key brain systems involved in emotional and cognitive regulation. The PFC, which continues to develop throughout adolescence, plays a critical role in executive functions, perspective shifting, goal-directed behavior, and the control of impulsive behavior. Thus, proper maturation of this brain region during adolescence is essential for developing appropriate information-processing pathways that support more complex behavioral outcomes (Caballero et al., 2016), including goal-directed cognitive processes and the synchronization of thoughts and actions (Blakemore and Choudhury, 2006; Best and Miller, 2010). The PFC is the last region of the brain to myelinate, and this process is key, as in adolescents it leads to the development of the ability to hypothesize, reason, think logically, socialize, weigh risks and rewards, and regulate mood (Dow-Edwards et al., 2019; Somerville et al., 2013).

Another system worth highlighting is the limbic system, which comprises a set of interrelated cortical and subcortical structures that link visceral states and emotions to cognition and behavior (Catani et al., 2013). Limbic regions such as the AMY and HIP mature earlier than cortical regions but remain highly responsive, contributing to heightened emotional sensitivity and reward-seeking behaviors. During adolescence, feelings and novel experiences are processed by areas of the limbic system, promoting its maturation and potentially leading to significant changes that persist beyond adolescence, underscoring the importance of this developmental phase (Cressman et al., 2010; Fuster, 2001; Frere et al., 2020; Braun, 2011). The imbalance between these systems has been proposed to underlie characteristic adolescent behaviors, including increased impulsivity and risk-taking (Casey et al., 2011).

During adolescence, brain maturation is marked by significant changes in neurotransmitter systems, including dopaminergic and glutamatergic signaling (Cicchetti and Donald, 2006; Fuster, 2001). These systems mature at different rates (Dow-Edwards et al., 2019) and play a crucial role in the development of mature cognitive patterns (Cicchetti and Donald, 2006). Variations in the maturation of the mesolimbic-mesocortical dopaminergic system and the slower growth of descending cortical glutamatergic innervation may contribute to increased risky behavior and substance abuse during adolescence (O'Dell, 2009; Bossong and Niesink, 2010).

Overall, adolescence is a critical period of heightened neuroplasticity, during which the ongoing refinement of neuronal circuits enables the development of complex cognitive and emotional functions. This dynamic reorganization is influenced by environmental factors (Blakemore and Choudhury, 2006; Fuhrmann et al., 2015; Skaper et al., 2017), making this developmental window particularly sensitive to external influences that affect behavior and the onset of psychiatric disorders (Choudhury and McKinney, 2013).

In this context, exposure to stress during adolescence can profoundly affect the development and function of brain circuits, leading to behavioral problems that persist into adulthood. In humans, one of the most significant triggers of mental illness during adolescence is social stress (Fuhrmann et al., 2015; Corell et al., 2022), including peer-related adversity such as bullying. Suffering physical or psychological harm from peers negatively affects mental and physical wellbeing (Herge et al., 2016; Hasler et al., 2020) and is consistently associated with an increased risk of depressive symptoms in adolescence and later in life (Silk et al., 2014). Moreover, evidence indicates that exposure to stressors during adolescence increases the risk of developing schizophrenia (Lenroot and Giedd, 2010) and neuroticism (Metts et al., 2021).

In addition, cumulative stressors such as family conflict, academic difficulties, and social instability further contribute to long-term alterations in emotional regulation and behavioral outcomes (Kovacs-Balint et al., 2023). Interestingly, cumulative stress during late adolescence has been associated with an attenuated mPFC reward response in young adult men, which is linked to increased vulnerability to problematic alcohol use (Casement et al., 2015).

At the biological level, chronic exposure to stressful life events during adolescence alters HPA responsiveness, promoting sustained release of stress hormones and peripheral immune activation (Oldehinkel and Bouma, 2011). This, in turn, facilitates the recruitment of immune cells and the release of pro-inflammatory cytokines, which can influence brain function and contribute to neuroinflammation. Importantly, microglial activation and alterations in blood-brain barrier integrity have been proposed as key mechanisms that mediate the impact of peripheral immune signals on the brain, particularly in regions involved in emotional regulation.

These neurobiological alterations are associated with disrupted maturation of prefrontal-limbic connectivity, impaired top-down control of emotional responses, and increased behavioral reactivity. In addition, stress exposure during adolescence has been linked to long-lasting changes in reward processing and increased vulnerability to substance use and affective disorders. Likewise, consumption of psychoactive drugs during adolescence also disrupts synaptic reorganization and the maturation of brain circuits (Squeglia et al., 2009; Casey et al., 2008). One particularly vulnerable region is the PFC, which undergoes extensive myelination and synaptic pruning during this developmental period. Because the subcortical areas associated with reward and emotional regulation, such as the striatum and AMY, mature later than the executive and cognitive control mechanisms, psychoactive drugs exacerbate this developmental imbalance. Moreover, alterations in dopaminergic and stress-response systems have been associated with increased impulsivity and sensation-seeking, as well as cognitive and emotional alterations, including severe anxiety and depressive symptomatology, increasing the risk of developing psychiatric disorders and addictive behaviors in adulthood (Stankovic and Colak, 2022; Eiden et al., 2023; Karpova et al., 2021).

At the cognitive level, stress exposure during adolescence also influences information processing and emotional reactivity. Acute stress has been shown to enhance self-referential processing, particularly in individuals with increased vulnerability to depressive symptoms, potentially reinforcing negative cognitive biases. In contrast, positive feedback following stress exposure can engage neural circuits associated with reward, motivation, and attention, supporting adaptive learning and resilience (Celen et al., 2024). These findings highlight that the impact of stress during adolescence is not uniform but depends on individual susceptibility and the nature of environmental inputs.

Importantly, the effects of stress during adolescence are modulated by factors such as sex, age, and stressor type, highlighting the complexity of this developmental window. Bidirectional interactions between stress exposure and behavioral traits, such as irritability, further support the idea that adolescence represents a sensitive period during which environmental challenges can shape long-term emotional and cognitive outcomes (Harrison et al., 2025).

The ECS has emerged as a key modulator of brain development and of the regulation of stress and anxiety across the stages of life (Meyer et al., 2018). During adolescence, changes in the ECS occur in parallel with the refinement and maturation of brain regions, especially within the corticolimbic circuit. A central function of the ECS is modulating the excitatory/inhibitory balance, which contributes to stabilizing neural network activity and coordinating communication between brain regions such as the PFC, AMY, and HIP. Through these mechanisms, the ECS appears to support the functional integration of circuits involved in emotional regulation.

Interestingly, stress exposure during adolescence has been shown to alter ECS signaling, which may in turn contribute to disruptions in circuit function and behavioral outcomes, highlighting the ECS’s role as a mediator of stress effects during this developmental stage. Although most of the evidence comes from CB1r, additional studies suggest that CB2r may also participate in these processes, particularly through its association with neuroimmune pathways. Interestingly, adolescent drug exposure also alters the ECS, including the CB2r, which serves as a pivotal bridge between the endocannabinoid and neuroimmune systems (Garcia-Cabrerizo and Garcia-Fuster, 2016; Morcuende et al., 2022; Zamberletti et al., 2015).

### 3.2 Evidence for CB2r involvement in adolescent adversity-induced vulnerability

Although considerably less studied than ELS, growing evidence indicates that CB2r is also sensitive to environmental challenges during adolescence. Exposure to stressors and recreational drugs during this critical developmental window has been associated with long-lasting alterations in CB2r expression in brain regions involved in emotional and reward-related behaviors.

In particular, adolescent stress appears to modify CB2r expression in a brain-region-, sex-, and paradigm-dependent manner. Repeated tail-shock stress reduced CB2r protein expression in the PFC of both male and female adolescent rats, whereas females exhibited higher basal Cnr2 gene expression across several corticolimbic regions, highlighting sex-dependent differences in CB2r regulation during this developmental period (Xing et al., 2014). In contrast, both acute and repeated restraint stress increased amygdalar Cnr2 expression and induced persistent anxiety-like behaviors in adulthood (Sanchez-Marin et al., 2022a; Sanchez-Marin et al., 2022b). In the acute stress paradigm, these changes were accompanied by elevated plasma corticosterone, increased circulating 2-AG levels, and alterations in glutamatergic signaling, whereas repeated restraint stress induced broader changes in ECS-related genes, together with changes in glutamatergic and CRH signaling. Additional evidence indicates that adolescent stress also modifies the response of CB2r to subsequent environmental insults. In a model combining repeated restraint stress with intermittent alcohol exposure, prior stress prevented alcohol-induced reductions in hippocampal Cnr2 expression, along with other ECS- and glutamate-related genes, partially restoring hippocampal molecular homeostasis and attenuating long-term memory deficits (Verheul-Campos et al., 2025).

Similarly, exposure to recreational drugs during adolescence has also been associated with alterations in CB2r expression. Cocaine (Garcia-Cabrerizo and Garcia-Fuster, 2016) and alcohol (Guo et al., 2022) consumption reduced CB2r protein levels in the PFC and HIP, respectively. Interestingly, the direction of these changes has been reported to depend on the experimental paradigm, as chronic alcohol consumption during late adolescence increased CB2r expression in the PFC (Li et al., 2023). Notably, these alterations were accompanied by neuroimmune changes. Chronic alcohol consumption during late adolescence increased NLRP3 inflammasome activation in the PFC and promoted microglial activation and a less ramified morphology, changes associated with increased anxiety-like behaviors (Li et al., 2023). Likewise, adolescent intermittent alcohol consumption induced cognitive deficits accompanied by microglial activation and a shift toward a pro-inflammatory neuroimmune profile in the HIP (Guo et al., 2022). In addition, adolescent exposure to JWH-018, the main compound of the synthetic drug Spice/K2, induced persistent deficits in sensorimotor gating and sex-dependent alterations in anxiety-like behaviors, accompanied by reduced CB2r expression in the PFC, activation of microglia and astrocytes in the PFC, and reduced GAD67 expression (Izquierdo-Luengo et al., 2023).

Pharmacological manipulation of CB2r further supports its involvement in drug-related behavioral and neurobiological processes during adolescence. In this respect, the selective CB2r agonist AM1241 has been shown to prevent alcohol-induced neuroinflammatory changes in the PFC and anxiety-like behaviors during withdrawal (Li et al., 2023). Moreover, direct pharmacological modulation of CB2r during adolescence has been shown to produce long-lasting alterations in reward-related learning. Administration of the CB2r antagonist/inverse agonist SR144528 during adolescence impaired the acquisition of Pavlovian conditioned responses in adulthood, without affecting instrumental conditioning. These findings suggest that CB2r activity during adolescence contributes to the development of specific aspects of reward-related behavior (Ellner et al., 2021). Together, these pharmacological studies provide evidence that CB2r contributes to long-term behavioral consequences of adolescent environmental insults, supporting this receptor as a potential target for interventions aimed at mitigating stress- and drug-induced consequences.

In summary, these studies indicate that adverse experiences during adolescence are associated with alterations in CB2r that appear to vary by the nature of the exposure, brain region, sex, and timing within the developmental period. These changes are accompanied by emotional, cognitive, and neuroimmune alterations. Importantly, although limited, pharmacological evidence suggests a promising role for this receptor in shaping vulnerability to adverse experiences during adolescence (Figure 2, Table 2).

**FIGURE 2:**
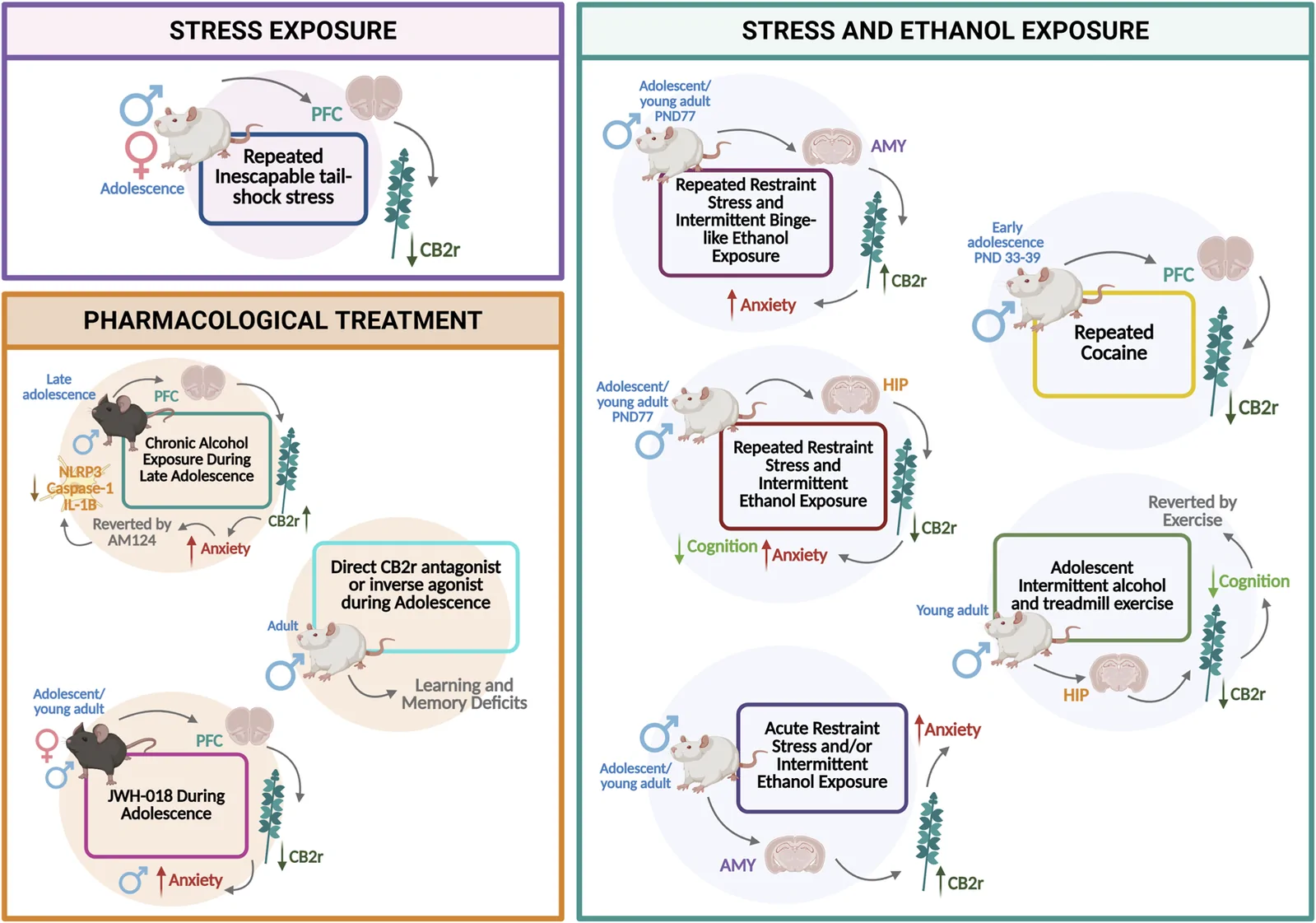
Schematic representation of the main findings supporting the involvement of CB2r in the consequences of adverse experiences during adolescence. The figure summarizes the main alterations in CB2r reported following stress and drug exposure during adolescence, including brain region- and sex-dependent differences and the associated behavioral and neurobiological outcomes. The effects of pharmacological manipulation of CB2r on these alterations are also represented. HIP: hippocampus; AMY: amygdala; NAcc: nucleus Accumbens; PFC: prefrontal cortex. ↑ and ↓ indicate increased and decreased expression, respectively.

**TABLE 2:** CB2r receptor alterations and functional evidence following adolescent adversity.

Model	Species/Strain	Sex	Developmental period/Assessment	Brain region	CB2r-related findings	Behavioral outcomes	Pharmacological/Genetic manipulation	Molecular outcomes	Behavioral outcomes	Refs.
Repeated inescapable tail-shock stress (2h/day, 3 days)	Rat/Sprague Dawley	M/F	Adolescence; assessed after repeated stress	PFC, AMY, HIP and hypothalamus	↓ CB2r in the PFC in M and F	_	_	_	_	Xing et al. (2014)
Acute restraint stress and/or intermittent alcohol exposure during adolescence	Rat/Wistar	M	Adolescence/young adult	AMY	↑ Cnr2	Anxiety-like behaviors	_	_	_	Sanchez-Marin et al. (2022a)
Repeated restraint stress and intermittent binge-like alcohol exposure	Rat/Wistar	M	Adolescence/adult assessment at PND77	AMY, mPFC	↑ Cnr2 in AMY	Anxiety-like behaviors	_	_	_	Sanchez-Marin et al. (2022b)
Repeated restraint stress (PND32-36) followed by intensive intermittent alcohol exposure	Rat/Wistar	M	Adolescence/adult assessment at PND77	HIP	Alcohol ↓ Cnr2 in HIP; prior RS prevented/restored this reduction	Alcohol impaired recognition memory and increased anxiety-like behavior; prior stress prevented the memory deficit, but not anxiety	_	_	_	Verheul-Campos et al. (2025)
Repeated cocaine (15 mg/kg/day; 7 days)	Rat/Sprague- Dawley	M	Early (PND33-39), mid (PND40-60) or late adolescence (PND47-53); adult follow-up at PND64	PFC, HIP	↓ Basal CB2r across adolescence Cocaine during early adolescence ↓ CB2r in PFC	_	_	_	_	Garcia-Cabrerizo and Garcia-Fuster (2016)
Adolescent intermittent alcohol (AIE) + treadmill exercise	Rat/Sprague- Dawley	M	Adolescent ethanol exposure; young-adult assessment	HIP	AIE ↓ CB2r in HIP; treadmill exercise further ↓ CB2r	AIE induced learning and memory deficits; exercise improved cognitive performance	_	_	_	Guo et al. (2022)
Chronic alcohol exposure during late adolescence	Mouse/C57BL/6J	M	Late adolescence; withdrawal assessed 24 h after last alcohol exposure	PFC	↑ CB2r	Anxiety-like behaviors	Selective CB2r agonist AM1241 (3 and 6 mg/kg; i.p.; PND42-PND70)	Ameliorated anxiety-like behaviors	Attenuated microglia and NLPR3/caspase-1/IL-1β signaling activation	Li et al. (2023)
JWH-018 (0.5, 1 and 1.5 mg/kg, i.p.) exposure during adolescence (PND35-49)	Mouse/C57BL/6J	M/F	Short- and long-term assessment	PFC	Transient ↓ CB2r in M PFC	Sex- and time-dependent anxiety alterations; persistent sensorimotor gating deficits in M	_	_	_	Izquierdo-Luengo et al. (2023)
Direct CB2r antagonism/inverse agonism during adolescence	Rat/Sprague- Dawley	M	SR144528 (3.2 and 6.4 mg/kg; i.p.) from PND28-41; behavior assessed in adulthood	Mesocorticolimbic/reward circuit inferred	_	Adolescent CB2r inhibition impaired acquisition of Pavlovian sign-tracking in adulthood	_	_	_	Ellner et al. (2021)

PND, postnatal day; AEI, adolescence intermittent alcohol; M, male; F, female; PFC, prefrontal cortex; HIP, hippocampus; AMY, amydgala; mPFC, medial prefrontal cortex; RS, restraint stress.

## 4 Current gaps, future perspectives and conclusions

The available evidence suggests that CB2r may be involved in the behavioral and molecular consequences of adverse experiences during early development. Current studies have reported alterations in CB2r expression in corticolimbic brain regions following exposure to stressors in postnatal life and early adolescence. This change is dynamic across development and appears to depend on sex and the brain region analyzed. Moreover, the few pharmacological studies carried out to date also suggest a role for CB2r, at least in part, in the behavioral and molecular consequences of early-life and adolescent stress. Available evidence suggests that activating CB2r may mitigate neuroimmune changes associated with early adversity, primarily by modulating microglial inflammatory responses (Figure 3).

**FIGURE 3:**
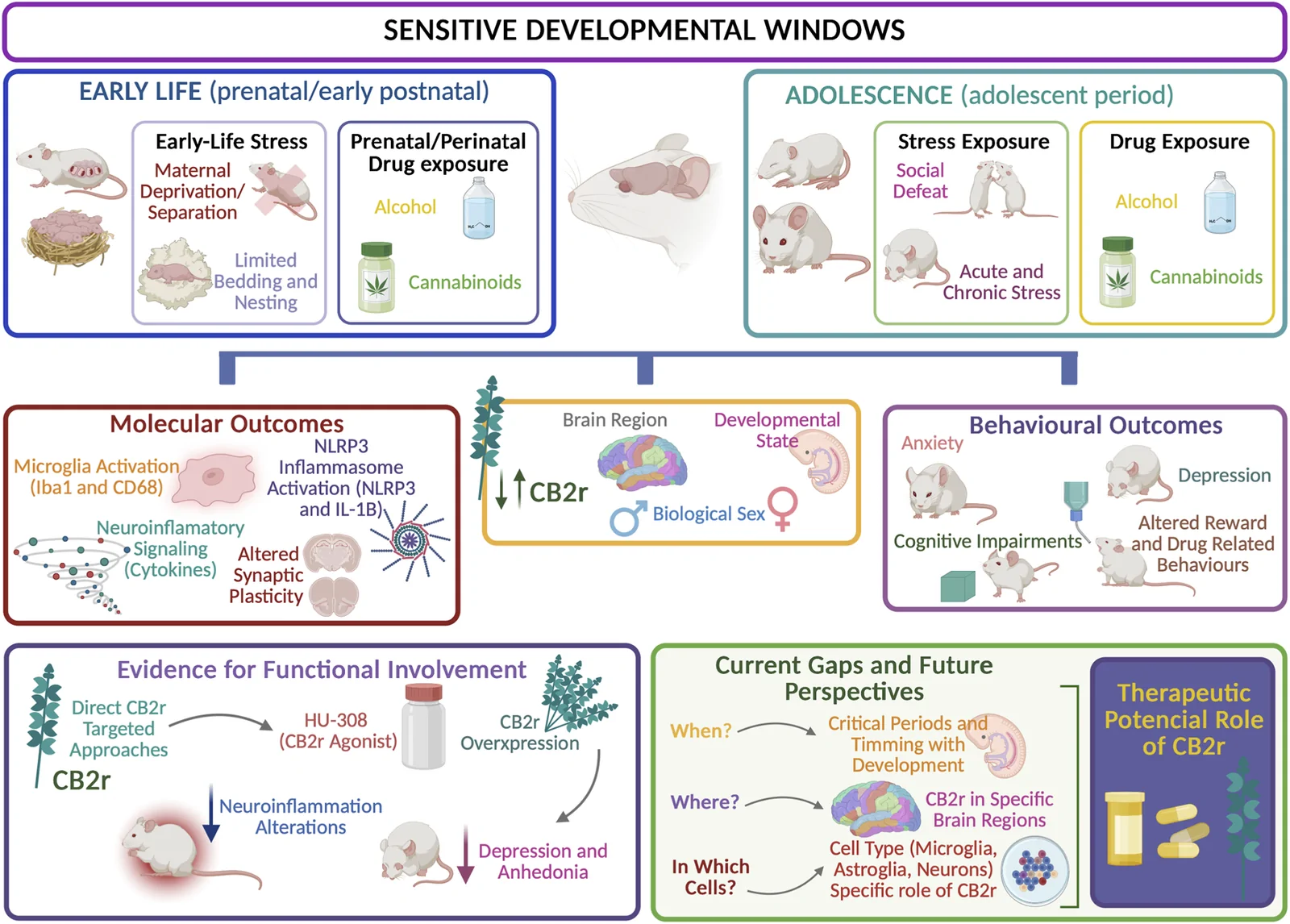
Proposed framework for the involvement of CB2r in developmental adversity and affective vulnerability. Adverse experiences during sensitive developmental windows, including early life and adolescence, are associated with dynamic alterations in CB2r expression and signaling. These alterations depend on developmental stage, biological sex, brain region, and the nature of the adverse experience, and occur alongside neuroimmune, molecular, and behavioral alterations. Available pharmacological and genetic evidence further suggests that modulation of CB2r can influence some of these outcomes. However, the precise contribution of CB2r depends on the developmental and cellular context and remains to be fully established. Further studies defining when, where, and in which cellular populations CB2r signaling contributes to these responses will be essential to determine its therapeutic potential. ↑ and ↓ indicate increased and decreased expression, respectively.

Despite these promising findings, evidence remains limited. Heterogeneity across experimental models, including differences in the nature, duration, and intensity of the stressor, as well as in the developmental stage and brain regions evaluated, makes comparisons across studies difficult and hampers the possibility of establishing consistent conclusions. To address this heterogeneity, a useful approach would be to systematically compare key experimental variables within the same or closely comparable design. For example, different ELS paradigms, such as maternal separation/deprivation and limited bedding and nesting, should be compared at equivalent developmental stages to determine whether CB2r depends on the nature and characteristics of the adverse experience. Since corticolimbic brain regions are closely related to emotional responses, parallel assessment of these brain regions, particularly PFC, HIP and AMY, is needed to understand the regional specificity of CB2r alterations. Furthermore, greater consistency in the behavioral traits evaluated, including affective and cognitive outcomes, would further facilitate comparisons across studies. Moreover, future longitudinal studies evaluating CB2r at multiple time points following the same paradigm will be essential for characterizing its dynamic adaptations from early life to adolescence and adulthood, and for distinguishing transient from persistent alterations.

Determining whether CB2r alterations occur in neurons, microglia, and/or astrocytes will be key to understanding its role in the consequences of developmental adversity (Figure 3). Changes in CB2r expression at the tissue level do not provide information about the specific cellular population in which these adaptations occur. This distinction may be particularly relevant for achieving a more precise understanding of the role of CB2r in specific cellular populations. For example, cell-specific approaches, such as microglia-specific conditional CB2r knockout models, could provide further insight into this aspect.

Biological sex may also influence CB2r changes induced by developmental adversity. However, relatively few studies have directly compared males and females under the same experimental conditions. Therefore, the current evidence is insufficient to identify consistent sex-dependent patterns, highlighting the need for future studies specifically designed to address this question. Both sexes should be evaluated under the same experimental conditions to determine whether CB2r changes are sex-dependent.

Importantly, changes in Cnr2 or CB2r protein expression do not necessarily reflect changes in receptor activity or downstream signaling. Thus, further functional studies, e.g., using inducible conditional knockout models, could help determine whether CB2r contributes to the behavioral and neurobiological consequences of developmental adversity or attenuates these effects. Similarly, local pharmacological manipulation or CB2r-targeting knockdown approaches (e.g., siRNA-mediated CB2r knockdown or CB2r-targeting antisense oligonucleotides) in brain regions such as the HIP could help establish the region-specific contribution of CB2r. Additionally, the available findings indicate that the behavioral and molecular brain alterations associated with adverse experiences during early life and adolescence do not depend solely on CB2r. Rather, the alterations reviewed here likely occur within broader ECS adaptations rather than representing an exclusively CB2r-mediated response. Further functional studies are therefore required to establish the specific contribution of CB2r and its interaction with other components of the ECS and with molecular systems involved in stress-related vulnerability.

As highlighted in the present review, only a limited number of studies have evaluated the functional role of CB2r using pharmacological approaches. Nevertheless, these studies provide evidence that CB2r agonism attenuates behavioral and neurobiological alterations, particularly those involving neuroimmune mechanisms. The timing of CB2r modulation is also an important question for future research. Because most pharmacological studies have examined the effects of CB2r modulation during or shortly after exposure to early-life and adolescent adversity, future studies should evaluate the efficacy of CB2r agonists, such as HU-308 or AM1241, administered at different time points, including later stages when the consequences of adversity may already be established (Figure 3). Such studies would help determine whether CB2r activation may modulate short-term alterations and persistent consequences emerging during late adolescence or young adulthood and would help define the therapeutic window for CB2r modulation.

Translating these findings to humans will require substantially more evidence on the functional and therapeutic relevance of CB2r. An initial translational approach could be to investigate whether CB2r-related alterations can also be detected in peripheral immune cells from children or adolescents exposed to early-life adversity. For example, CNR2 expression in lymphocytes could be explored as a minimally invasive measure and examined longitudinally in relation to clinical outcomes or response to interventions, such as cognitive behavioral therapy. Thus, future clinical and translational studies will be necessary to determine whether the developmental alterations in CB2r observed in experimental models are also present in humans and to establish their relevance to stress-related psychiatric disorders. An additional translational limitation is that the types of adversity examined in humans and in the preclinical CB2r literature are not directly equivalent. While human studies frequently focus on social adversity during adolescence, including bullying and negative peer relationships, preclinical studies investigating CB2r have mainly used paradigms such as inescapable tail shock or acute/repeated restraint stress, as well as drug exposure. Thus, whether CB2r-related adaptations also occur in response to adverse social experiences remains largely unexplored. Future studies using preclinical models that capture specific components of adolescent social adversity could help bridge this translational gap.

Overall, adversity during sensitive developmental windows is associated with persistent and dynamic alterations in CB2r that appear to vary according to developmental stage, biological sex, and brain region. These changes occur alongside affective, behavioral, and neuroimmune alterations, while the available pharmacological and genetic evidence suggests that CB2r may contribute to the behavioral and neurobiological consequences of developmental adversity. Further studies integrating pharmacological and genetic approaches will be needed to establish the functional relevance of these alterations and to determine how factors such as developmental timing, biological sex, brain region, and cellular population influence CB2r responses to developmental adversity. Addressing these questions will be important to determine whether CB2r modulation may have therapeutic relevance.
